# Fast Tac Metabolizers at Risk—It is Time for a C/D Ratio Calculation

**DOI:** 10.3390/jcm8050587

**Published:** 2019-04-28

**Authors:** Katharina Schütte-Nütgen, Gerold Thölking, Julia Steinke, Hermann Pavenstädt, René Schmidt, Barbara Suwelack, Stefan Reuter

**Affiliations:** 1 Department of Medicine D, Division of General Internal Medicine, Nephrology and Rheumatology, University Hospital of Münster, 48149 Münster, Germany; katharina.schuette-nuetgen@gmx.de (K.S.-N.); gerold.thoelking@ukmuenster.de (G.T.); j_steinke@ymail.com (J.S.); hermann.pavenstaedt@ukmuenster.de (H.P.); barbara.suwelack@ukmuenster.de (B.S.); 2 Institute of Biostatistics and Clinical Research, University Hospital of Münster, 48149 Münster, Germany; rene.schmidt@ukmuenster.de

**Keywords:** kidney transplantation, tacrolimus, C/D-ratio, pharmacokinetics

## Abstract

Tacrolimus (Tac) is a part of the standard immunosuppressive regimen after renal transplantation (RTx). However, its metabolism rate is highly variable. A fast Tac metabolism rate, defined by the Tac blood trough concentration (C) divided by the daily dose (D), is associated with inferior renal function after RTx. Therefore, we hypothesize that the Tac metabolism rate impacts patient and graft survival after RTx. We analyzed all patients who received a RTx between January 2007 and December 2012 and were initially treated with an immunosuppressive regimen containing Tac (Prograf^®^), mycophenolate mofetil, prednisolone and induction therapy. Patients with a Tac C/D ratio <1.05 ng/mL × 1/mg at three months after RTx were characterized as fast metabolizers and those with a C/D ratio ≥1.05 ng/mL × 1/mg as slow metabolizers. Five-year patient and overall graft survival were noticeably reduced in fast metabolizers. Further, fast metabolizers showed a faster decline of eGFR (estimated glomerular filtration rate) within five years after RTx and a higher rejection rate compared to slow metabolizers. Calculation of the Tac C/D ratio three months after RTx may assist physicians in their daily clinical routine to identify Tac-treated patients at risk for the development of inferior graft function, acute rejections, or even higher mortality.

## 1. Introduction

Tacrolimus (Tac) is recommended by The Kidney Disease: Improving Global Outcomes (KDIGO) guideline as the immunosuppressant of choice after renal transplantation (RTx) [1]. Although it is very effective in terms of preventing organ rejection, its highly inter-individual variable metabolism rate can be a challenging factor for physicians as many factors can impact on Tac metabolism [2]. Different approaches have largely failed to predict the dosing and Tac clearance or could not show the advantages pertaining to safety or outcomes [3,4,5]. Even though genetic polymorphisms have been shown to significantly influence Tac metabolism, genetic testing strategies did not improve clinical outcomes [6,7], and require effort in terms of cost and the interpretation of results and therefore have not found their way into clinical practice yet. Thus, therapeutic drug monitoring is essential for directing the therapy.

We recently proposed a classification of patients receiving Tac into two major metabolism groups. Our stratification is based on the calculation of the C/D ratio (expressed as the trough level concentration normalized by the dose). A C/D ratio <1.05 ng/mL × 1/mg identifies fast metabolizers, whereas a C/D ratio ≥1.05 ng/mL × 1/mg indicates a slow metabolism [8]. Alternative definitions of the metabolic state category, such as dose requirements [9], clearance rate, or calculation of the D/C ratio, exist [10,11]. Interestingly, fast Tac metabolizers have been found as being more prone to developing BK viremia [12], calcineurin-inhibitor toxicity [8,9], and acute rejections [10,13,14] after RTx. In congruence, kidney function (three to 24 months after RTx and 36 months after liver transplantation, respectively) was lower in fast than in slow metabolizers [8,9,15,16]. Based on these findings, suggesting an influence of fast Tac metabolism on adverse events and inferior renal function after renal transplantation, the aim of this study was to analyze whether Tac metabolism type might even impact on definite outcomes such as patient and graft survival and to identify whether fast Tac metabolism constitutes an independent risk factor that physicians should consider besides already known determinants of kidney transplant patients’ long-term outcome. Hypothesizing that Tac metabolism-dependent effects on mortality might become discernable in the long-term, the present study was performed in a patient cohort with a complete five-year follow-up. 

## 2. Methods

### 2.1. Patients

Prior to analysis, all patient data was anonymized and de-identified. The local ethics committee (Ethik Kommission der Ärztekammer Westfalen-Lippe und der Medizinischen Fakultät der Westfälischen Wilhelms-Universität, No. 2014-381-f-N) approved the study. The methods used in this study were carried out in accordance with the current transplantation guidelines and the Declarations of Istanbul and Helsinki. Written informed consent with regard to recording their clinical data was given by all participants at the time of transplantation.

We retrospectively analyzed all patients who underwent RTx between January 2007 and December 2012 at the University Hospital Münster and were initially treated with an immunosuppressive regimen containing Tac (Prograf^®^), mycophenolate mofetil, prednisolone, and induction therapy. Oral CMV-prophylaxis with valganciclovir was administered for 100 days for D+/R+, D−/R+ and D+/R− recipients, and none if both the donor and the recipient were negative for CMV. Recipients aged < 18 years, with combined transplants, and for whom the three month C/D ratio could not be adequately calculated (due to Tac-free immunosuppressive regimen, missing data, or simultaneous higher dosage of prednisolone (≥20 mg/day, which is known to induce CYP3A activity)) were excluded. The Tac target trough level was 6–10 ng/mL. Recipient and donor data was collected from the patient files. The following parameters were examined: Patient and donor demographics, recipient body mass index (BMI), recipient history of hypertension or diabetes mellitus, cause of end-stage renal disease (ESRD), number of prior kidney transplants, time on dialysis, donor type of transplantation, degree of human leukocyte antigen (HLA)-mismatching, current panel-reactive antibodies (PRA), cold and warm ischemia time and incidence of new-onset diabetes after transplantation (NODAT) and cytomegalovirus (CMV) DNAaemia (a number of >600 copies/mL was considered as relevant corresponding to the threshold value given by the manufacturer (TaqMan-PCR, QIAamp DNA Blood Kit, Qiagen, Hilden, Germany)). CMV screening was performed monthly during the first six months after RTx, every second month during months 6–12, and on indication.

### 2.2. Tacrolimus Metabolism Rate

Tac metabolism rates were calculated at three months after RTx by dividing the Tac blood trough concentration (C) by the corresponding daily Tac dose (D), as published before [8,16].

C/D ratio (ng/mL × 1/mg) = blood Tac trough concentration (ng/mL)/daily Tac dose (mg)(1)

As inpatient values are more prone to errors due to coexisting factors like diarrhea, anaemia and CYP3A4 interfering drugs as azoles, e.g., only outpatient tacrolimus concentrations were considered. Measurements with exceptional high Tac trough concentrations (>15 ng/mL) were not considered to exclude false-high values due to Tac ingestion prior blood sampling. 

For 50 randomly selected patients we additionally calculated the Tac C/D ratio at one and six months as an average C/D ratio and compared it to the three-month C/D ratio to account for further potential factors that can influence the C/D ratio and might affect single-time point measurements. As the 3-month C/D ratio strongly correlated with the average C/D ratio at month one and six, we applied the 3-month C/D ratio for the following patient categorization: 

As defined previously, patients with a Tac C/D ratio <1.05 ng/mL × 1/mg were categorized as fast metabolizers. Patients with a C/D ratio of 1.05–1.54 ng/mL × 1/mg or a C/D ratio ≥1.55 ng/mL × 1/mg were defined as intermediate metabolizers and slow metabolizers, respectively [8]. For simplification, intermediate and slow metabolizers were summarized as slower metabolizers in this study.

### 2.3. Outcome Measures

The main outcome measures were patient and overall graft survival. Patient survival was defined as time from RTx to death (from any cause) or last contact for alive patients. Overall graft survival was defined as the time from RTx to death (from any cause), graft failure, or last contact, whichever occurred first. Graft failure was defined as the reinitiation of dialysis treatment. 

Further outcome parameters were serum creatinine and estimated glomerular filtration rate (eGFR) at years one to five after transplantation as well as the frequency of biopsy-proven acute rejection episodes (defined by Banff classification) and the rejection-free survival. Patients were subjected to kidney biopsy in case of a relevant rise in creatinine (≥0.3 mg/dL). Biopsies were evaluated by two pathologists. 

Whole blood was analyzed for creatinine (enzymatic assay; Creatinine-Pap, Roche Diagnostics, Mannheim, Germany) and Tac (automated tacrolimus (TACR) assay; Dimension Clinical Chemistry System; Siemens Healthcare Diagnostic GmbH; Eschborn; Germany). Only 12 h Tac trough levels were used for analysis. Renal function was determined by calculating the eGFR using the CKD-EPI equation.

### 2.4. Statistical Analysis

Statistical analysis was performed using IBM SPSS^®^ Statistics 25 for Windows (IBM Corporation, Somers, NY, USA). Normally distributed continuous variables are shown as mean ± standard deviation (SD) and non-normally distributed continuous variables as median and 1st and 3rd quartiles (interquartile range, IQR). Absolute and relative frequencies have been given for categorical variables. Pairs of independent groups were compared using the Student’s t-test for normally distributed data, Mann–Whitney U test for non-normal data, and Fisher’s exact test for categorical variables. To compare paired data, we used the Wilcoxon test for continuous variables and the McNemar test for categorical variables. 

Survival analyses were based on a maximum follow-up of five years after RTx. Patient survival, overall allograft survival as well as rejection-free survival were analyzed using the Kaplan-Meier method [17], and the groups were compared using the log-rank test. Cox proportional hazards regression models [18] were built using a stepwise variable selection procedure to assess the association between C/D ratio metabolism status and survival while simultaneously adjusting for potential confounding factors (inclusion: *p*-value of the score test ≤ 0.05, exclusion: *p*-value of the likelihood ratio test > 0.1). Results have been presented as hazard ratios (HR) with 95% confidence interval (95% CI) and *p*-value of likelihood ratio test. The *p*-value of score test is given for non-selected variables in multivariable analyses.

Mixed models with AR (1) covariance structure were fitted to analyze the impact of biological and clinical markers on the time course of eGFR between year one and five after the transplantation based on the eGFR values observed at annual intervals during this period. Univariable analyses included each marker separately along with its interaction with time since baseline measurement (at year one after transplantation) in order to assess (i) the baseline eGFR and/or (ii) whether potential time trends of eGFR differ between the subgroups defined by the marker. Multivariable models were built using a stepwise variable selection procedure in order to assess the impact of C/D ratio metabolism status on baseline eGFR and time trends of eGFR while adjusting for potential confounding factors. Models included (i) C/D ratio metabolism status and its interaction with time since baseline measurement in a first block and (ii) potential confounding factors along with their interactions with time since baseline measurement in a second block with forwards variable selection (inclusion/exclusion criterion: *p*-value of Wald test ≤0.05/>0.1).

No adjustment for multiple testing was made, and all analyses were regarded as explorative. *p*-values ≤ 0.05 were considered statistically noticeable.

## 3. Results

### 3.1. Patient Cohort

**Figure 1:**
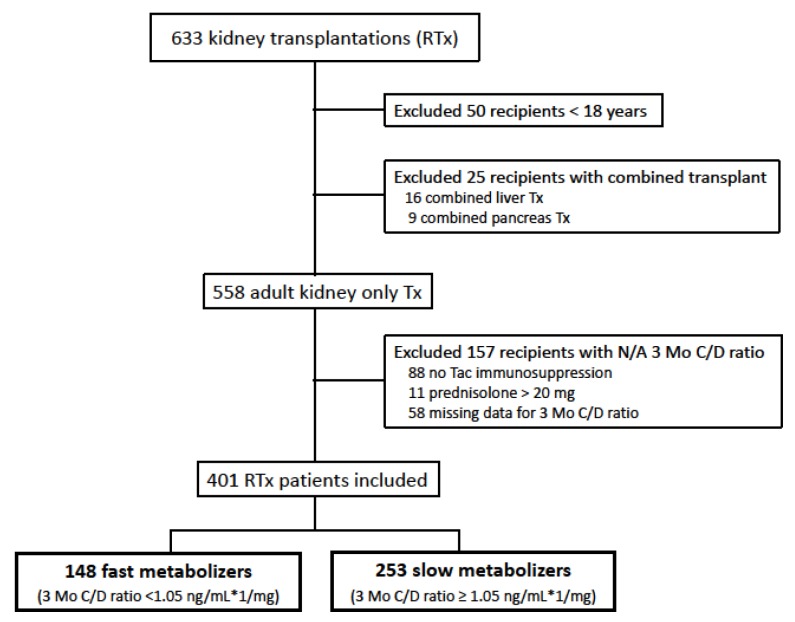
Enrollment flow chart for the study population. RTx = Renal transplantation; N/A: not available.

The enrollment flow chart for the study population is shown in Figure 1. Between January 2007 and December 2012, 633 kidney transplants were performed at our center. After the exclusion of 50 patients aged <18 years and 25 patients with combined transplantation, data on immunosuppressive therapy was extracted from the remaining 558 adult kidney-only transplant recipients. From these, 401 patients with an initial Tac-based immunosuppressive therapy and complete data on the 3-month C/D ratio were included. From all patients, 253 recipients (63.1%) were categorized as slow metabolizers and 148 recipients (36.9%) as fast metabolizers. The average C/D ratio of month one and six for 50 randomly selected patients did not differ from the three-month C/D ratio (*p* = 0.765, Table S1) and categorization of slow and fast Tac metabolizers was similar when applying the three-month C/D ratio or the average C/D ratio of months one and six (*p* = 1.000, Table S2), suggesting that three-month C/D ratio strongly correlated with the average C/D ratio during months one and six. 

Baseline patient characteristics for donors and recipients and transplantation-associated parameters are shown in Table 1. Tac mean trough levels and daily doses were noticeably different between the groups. The two groups were similar with respect to all other baseline characteristics that were analyzed. 

### 3.2. Patient and Overall Allograft Survival

**Figure 2:**
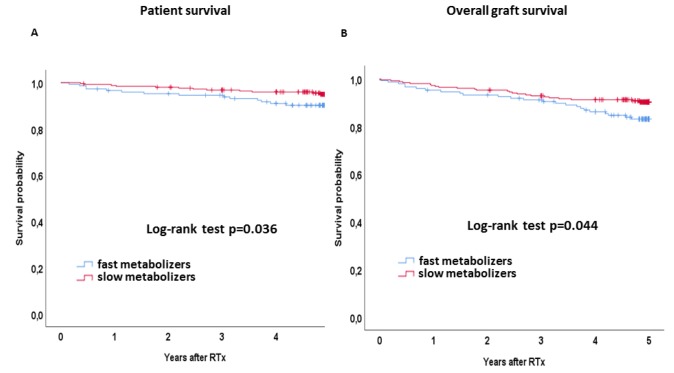
(**A**) Kaplan-Meier curves for patient survival and (**B**) overall graft survival. Survival rates of slow (red lines) and fast metabolizers (blue lines) were analyzed by the Kaplan–Meier method and compared using the log-rank test. Fast metabolizers showed a noticeably reduced patient and overall graft survival.

Kaplan-Meier curves for patient and overall allograft survival by Tac metabolism status are shown in Figure 2. Five-year patient survival was noticeably reduced in fast metabolizers as compared to slow metabolizers (89.9% vs. 95.3%, log-rank *p* = 0.036, Figure 2). The Cox regression analysis revealed a noticeable association between a fast Tac metabolism and patient survival in both univariable (HR 2.209 (95% CI 1.034–4.719), *p* = 0.041) as well as multivariable analysis (HR 5.749 (95% CI 1.556–21.242), *p* = 0.004) (Table 2). Overall allograft survival was affected by the Tac metabolism status as well: Fast metabolizers showed a noticeably reduced 5-year allograft survival rate as compared to slow metabolizers (83.8% vs. 90.5%, log-rank *p* = 0.044, Figure 2). HR was 1.772 (95% CI 1.006–3.121, *p* = 0.047)) for fast metabolizers in univariable Cox regression and 2.715 (95% CI 1.231–5.989, *p* = 0.012) after adjustment for potential confounders (Table 3). 

Causes of death are given in Table 4. While fast metabolizers mostly died from cardiovascular diseases (40%), the most common cause of death in slow metabolizers were infectious diseases (41.7%). In summary, a fast Tac metabolism noticeably affects patient as well as overall allograft survival after kidney transplantation. 

### 3.3. Renal Function

**Figure 3:**
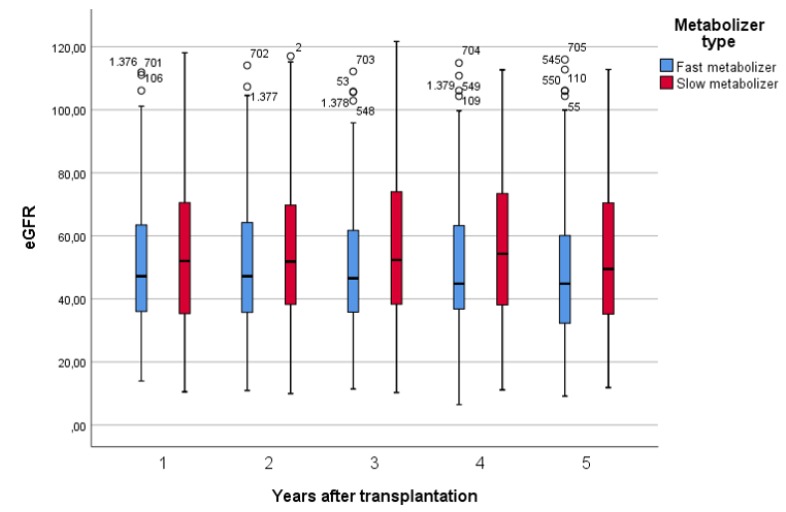
Time course of the eGFR within five years after renal transplantation. Fast metabolizers show a faster decline in the eGFR as compared to slow metabolizers over the first five years.

Renal function was assessed yearly within the first five years after transplantation. Figure 3 shows the development of the eGFR between year one and five after renal transplantation in slow and fast metabolizers. A linear mixed model was applied to estimate the time-dependent course of eGFR. Fast metabolizers showed a noticeably faster decline of the eGFR within five years after transplantation as compared to slow metabolizers in both univariable (*p* = 0.040) and multivariable analysis (*p* = 0.032) (Table 5a,b).

### 3.4. Rejections

**Figure 4:**
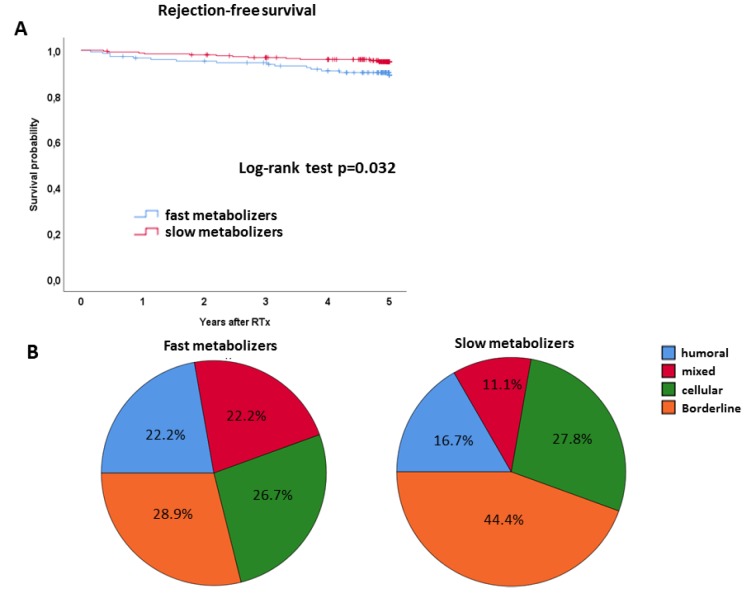
(**A**) Kaplan-Meier curves for rejection-free survival of slow (red lines) and fast metabolizers (blue lines), analyzed by the Kaplan–Meier method and compared using the log-rank test. Fast metabolizers showed a noticeably reduced rejection-free survival. (**B**) Subtype analysis of the first rejection episode within the first five years after transplantation. Fast metabolizers experienced increased frequencies of humoral and mixed acute rejection, whereas slow metabolizers were mainly affected by borderline rejections.

The Kaplan-Meier curve for rejection-free survival is shown in Figure 4A. The 5-year rejection-free survival was noticeably lower in fast metabolizers as compared to slow metabolizers (69.6% vs. 78.8%, log-rank *p* = 0.032, Figure 4A). The Cox regression analysis revealed a noticeable association between a fast Tac metabolism and rejection-free survival in univariable (HR 1.536 (95% CI 1.034–2.282), *p* = 0.035) as well as multivariable analysis (HR 1.622 (95% CI 1.085–2.424), *p* = 0.020) (Table 6). Table 7 shows the frequency of patients with ≥ 1 acute biopsy-proven rejection during the 5-year follow-up. While 45/148 (30.4%) fast metabolizers experienced at least one acute rejection, only 54/253 (21.3%) slow metabolizers were affected. Of note, the subtype analysis of the first rejection episode within the first five years after transplantation revealed an increased frequency of humoral and mixed rejections in fast metabolizers (*n* = 10, 6.8% vs. *n* = 9, 3.6% and *n* = 10, 6.8% vs. *n* = 6, 2.4%, respectively) (Table 7, Figure 4B), whereas slow metabolizers were mainly affected by borderline rejections. 

## 4. Discussion

Herein, we first described a significant influence of the Tac metabolism type on mortality after renal transplantation in a study population with a long-term observation period. A higher five-year mortality in fast metabolizers was accompanied by a higher rejection rate and inferior kidney function. Our study highlights the importance of a risk stratification strategy of RTx patients including information on individuals’ Tac metabolism rate which turned out to be an independent risk factor for a lower patient survival after renal transplantation. The C/D ratio is a simple tool that can be easily applied for this purpose. 

Based on our previous findings revealing an impact of fast Tac metabolism (C/D ratio <1.05 ng/mL×1/mg) on inferior renal function in a two-and three-year follow-up after RTx or LTx [8,16] we herein could demonstrate that this effect persists in the long term and that fast Tac metabolism also impacts on the time-dependent course of renal function in both univariable and multivariable analysis. Moreover, we identified fast Tac metabolism as an independent risk factor for a decreased graft survival. 

In congruence, Kuypers et al. observed that patients with high early Tac dose requirements (namely, fast metabolism) had a significantly reduced kidney function at three-months post-RTx [9]. This was attributed to an increased rate of calcineurin inhibitor (CNI)-related toxicity, which is in line with the observations of our previous study [8]. High-dose requirement in Kuyper’s study was associated with CYP3A5*1 genotype carriage in only 1/3 of cases, suggesting further factors impacting a patient’s Tac metabolism rate [2]. Notably, the area under the curve and the Tac trough level were not different between patients with and without CNI toxicity. The connection between different dose requirements and comparable trough levels in groups–although not calculated–hints at different C/D ratio categories of patients in both groups. Further, Genvigir et al. showed in a Brazilian cohort of CYP3A genotyped RTx patients that expression of CYP3A4/5 alleles leading to fast Tac metabolism (they also calculated the C/D ratio but did not calculate a cut-off) was associated with a lower eGFR at 3-months after RTx [15]. Again, no association was found between Tac exposure and the genetic score. By applying a multiple linear regression analysis, they showed that genetic variants and age impacted the C/D ratio. This is consistent with the literature – metabolism rate usually decreases with age – and with our findings that show tendencies of slow metabolizers being older age [8] (Table 1). Given the limitations of genetic testing-based strategies, we refrained from genotyping our patients but rather searched for a simple and cost-effective tool, as the C/D ratio, that can assist physicians in the daily routine to individualize their patients’ immunosuppressive therapy and stratify individuals with high risk for Tac-related side effects independent from complex genotyping-based methods.

In both aforementioned studies, rejection rates were calculated but not related to the C/D ratio or the dose requirements. However, as Kuypers et al. observed significantly higher rates of graft failure (32.3% vs. 13.7%) and lower rates of patients discontinuing steroids (5.8% vs. 23.7%) in patients requiring higher Tac doses, one can assume a higher rejection rate in these patients. We herein firstly describe a significant effect of the C/D ratio on acute rejections in a long-term follow up. In our study, rejection-free survival was increased in slow metabolizers, with higher frequencies of humoral and mixed rejections in fast than in slow metabolizers. In multivariable regression analysis, the BMI and the number of prior transplantations were associated with rejection as well. Recently, Barraclough et al. stated that the outcome of RTx patients depends on the immunosuppression within the first week after transplantation, although a relation between the AUC or Tac trough level and rejection was not detected [19]. Of note, as mentioned before, Tac AUC and trough levels are usually similar in slow and fast metabolizers and the C/D ratio was not calculated in their study. In a meta-analysis including data from the FDCC, Symphony, and OptiCept studies, Boumar et al. reported that the Tac trough concentration was not different between patients with and without acute rejection within the first 6 months after RTx [20]. Again, information regarding the Tac doses or the C/D ratio was not provided. In this regard, Egeland et al. observed that a high Tac clearance (or a fast metabolism) was associated with an increased risk of developing an acute rejection within the first few days after RTx [10]. Patients with a high Tac clearance might not reach the trough levels in time and suffer under-immunosuppression (at least at some time of the day). 

Mortality in fast metabolizers over the five-year observation period was consistently higher than in slow metabolizers, despite a tendency towards an older age in slow metabolizers. Overall, graft failure was low in both groups but aligned with the data from the literature [21,22]. In a recently published large registry analysis from England, the main reasons of death within the first year after RTX were stated as infection (21.6%), cardiovascular events (18.3%), and malignancy (7.4%) [21]. The main reasons of death in our cohort were cardiovascular diseases in fast metabolizers and infections in slow metabolizers, respectively, but did not differ between groups. Unfortunately, the reason of death remained unclear in 33.3% of cases in fast and 8.3% cases in slow metabolizers. As previously observed, fast metabolizers are more prone to developing BK virus infection than slow metabolizers. Thus, one can speculate that over-immunosuppression is an issue in these patients [12]. However, other infections, e.g., urinary tract infections, have not been shown to be related to the C/D ratio [23], and deaths due to infection were not different between groups in our cohort. This aligns with the fact that Tac mainly suppresses T-cell activity while the host’s defense to bacterial infections, which are more fatal in RTx patients than viral infections, is mainly based on innate immune cells [24]. Interestingly, 20% of death certificates in the English registry study stated “renal” as the cause of death within the first year after RTx [21]. Lastly, we were unable to identify a difference in reasons of death between groups. One reason for this could be the low mortality rate. However, factors that have been previously associated with increased risk of death, such as age at transplantation, diabetes, time on dialysis, or postmortal donation were not different between groups but rather distributed in favor of the fast metabolizer group (Table 1). Patient demographics associated with kidney function after RTx, such as living donation, number of transplants, cold ischemia time, hypertension, diabetes, donor age, and gender, did not differ between groups. This implies that the differences in renal function are likely to be related to Tac metabolism and rejection. Consequently, an inferior renal function is associated with higher mortality as cardiovascular events, infections as well as malignancies are related to kidney function [25].

We recognize that a study of this nature has limitations because of its retrospective design and potential errors inherent to maintaining a single-center database. Moreover, due to the relatively small patient size, inaccuracies in the data collection might affect the results; though data acquisition was performed thoroughly to avoid inconsistency or entry errors. The analyses are based on the assumption that coding errors and missing data are stochastic. Although we attempted to include as many relevant confounding parameters as possible there might still be residual factors that were not accounted for like the non-adherence of patients for example, which is difficult to measure. Prospective studies are needed to confirm our findings. We conclude from our data that the calculation of the C/D ratio, as a simple, cost-effective tool, can assist physicians in their daily clinical routine to identify Tac-treated patients at risk of developing an inferior graft function, acute rejections, or even higher mortality. This information should be used to individualize and optimize immunosuppressive therapy. 

## Figures and Tables

**Table 1:** Baseline patient characteristics.

	Slow Metabolizers (*n* = 253)	Fast Metabolizers (*n* = 148)	*p*-Value
Tac mean trough level at 3 months (ng/mL)	8.6 ± 2.8	7.1 ± 2.7	<0.001 ^a^
Tac daily dose at 3 months (mg/day)	4.9 ± 2.3	10.3 ± 4.3	<0.001 ^a^
Age (years, mean ± SD)	53.0 ± 13.4	50.2 ± 13.8	0.051 ^a^
Male sex, *n* (%)	156 (61.7)	80 (54.1)	0.142 ^c^
BMI (kg/m^2^, mean ± SD)	25.2 ± 4.0	25.2 ± 4.1	0.944 ^a^
Pre-existing recipient hypertension, *n* (%)	239 (94.5)	139 (94.6)	1.000 ^c^
Pre-existing recipient diabetes, *n* (%)	33 (13.0)	16 (10.9)	0.636 ^c^
Diagnosis of ESRD, *n* (%)			0.411 ^c^
Hypertension	20 (7.9)	11 (7.4)	
Diabetes	11 (4.3)	1 (0.7)	
Polycystic kidney disease	36 (14.2)	26 (17.6)	
Obstructive Nephropathy	20 (7.9)	14 (9.5)	
Glomerulonephritis	103 (40.7)	53 (35.8)	
FSGS	6 (2.4)	5 (3.4)	
Interstitial nephritis	4 (1.6)	2 (1.4)	
Vasculitis	5 (2.0)	2 (1.4)	
Other	45 (17.8)	34 (23.0)	
Time on dialysis (months, median (IQR))	60.5 (25.5, 90.3)	52.5 (24.9, 87.1)	0.323 ^b^
≥ 1 prior kidney transplant, *n* (%)	39 (15.4)	19 (12.8)	0.557 ^c^
Living donor transplantation	58 (22.9)	44 (29.7)	0.4 ^c^
Number HLA mismatch, *n* (%)			1.000 ^c^
0–3	169 (67.1)	98 (66.7)	
4–6	83 (32.9)	49 (33.3)	
Current PRA, *n* (%)			1.000 ^c^
0–20%	248 (98.0)	145 (98.0)	
> 20%	5 (2.0)	3 (2.0)	
Induction, *n* (%)			0.163 ^c^
Basiliximab	233 (92.1)	130 (87.8)	
Thymoglobulin	20 (7.9)	18 (12.2)	
Cold ischaemia time (hours, mean ± SD)	8.7 ± 4.9	8.2 ± 5.4	0.419 ^a^
Warm ischaemia time (min, mean ± SD)	31.8 ± 6.9	32.2 ± 8.0	0.684 ^a^
Donor age (years, mean ± SD)	53.4 ± 16.6	54.7 (13.7)	0.394 ^a^
Donor male sex, *n* (%)	121 (47.8)	63 (42.6)	0.350 ^c^

Demographic characteristics of the study population by the Tac metabolization status. Results are presented as mean ± standard deviation (SD) or median and first and third quartile (IQR), respectively, or as absolute and relative frequencies. BMI = body mass index; ESRD = end-stage renal disease; FSGS = focal segmental glomerulosclerosis; HLA = human leukocyte antigen; PRA = panel reactive antibodies. ^a^ Student’s *t*-test, ^b^ Mann-Whitney U test, ^c^ Fisher’s exact test.

**Table 2:** Univariable and multivariable analyses of patient survival using Cox regression.

Parameters	Univariable		Multivariable	
	HR (95% CI)	*p*-Value	HR (95% CI)	*p*-Value
Fast metabolizers vs. slow metabolizers (ref.)	2.209 (1.034–4.719)	0.041	5.749 (1.556–21.242)	0.004
Age (years)	1.057 (1.023–1.093)	0.001	-	0.081
Recipient sex Male vs. female (ref.)	1.631 (0.714–3.727)	0.246	-	0.262
Recipient BMI (kg/m^2^)	0.942 (0.852–1.042)	0.248	-	0.213
Pre-existing recipient hypertension yes vs. no (ref.)	1.512 (0.205–11.142)	0.685	-	0.635
Pre-existing recipient diabetes yes vs. no (ref.)	2.206 (0.890–5.468)	0.087	-	0.691
Cause of ESRD	-	0.852	-	0.738
Time on dialysis (months)	1.002 (0.993–1.011)	0.714	-	0.553
Prior kidney transplantation ≥1 vs. 0 (ref.)	1.379 (0.522–3.641)	0.517	-	0.707
Donor type Postmortal vs. living donor (ref.)	2.832 (0.853–9.405)	0.089	-	0.936
Number HLA mismatch 4–6 vs. 0–3	2.335 (1.097–4.968)	0.028	-	0.053
Current PRA >20% vs. 0–20%	1.951 (0.265–14.387)	0.512	-	0.709
Cold ischemia time (hours)	1.042 (0.972–1.118)	0.245	-	0.668
Donor age (years)	1.043 (1.014–1.074)	0.004	-	0.540
Donor sex Male vs. female (ref.)	0.928 (0.434–1.982)	0.847	-	0.266
NODAT yes vs. no (ref.)	2.983 (1.396–6.373)	0.005	5.150 (1.550–17.110)	0.005
CMV DNAaemia yes vs. no (ref.)	0.832 (0.352–1.968)	0.676	-	0.629
Acute rejection within 1 year yes vs. no (ref.)	1.610 (0.680–3.807)	0.279	-	0.947
eGFR at month 3 (mL/min/1.73m^2^)	0.979 (0.960–0.998)	0.028	-	0.999
eGFR at month 12 (mL/min/1.73m^2^)	0.968 (0.937–1.000)	0.047	-	0.166

Results are presented as hazard ratios (HR) with their 95% confidence interval (CI) and *p*-value of likelihood ratio test. For non-selected variables in multivariable analyses, *p*-value of score test is given. HR = hazard ratio; CI = confidence interval.

**Table 3:** Univariable and multivariable analyses of overall graft survival using Cox regression.

Parameters	Univariable		*p*-Value	
	HR (95% CI)	*p*-Value	HR (95% CI)	*p*-Value
Fast metabolizers vs. slow metabolizers (ref.)	1.772 (1.006–3.121)	0.047	2.715 (1.231–5.989)	0.012
Age (years)	1.056 (1.030–1.082)	<0.001	-	0.673
Recipient sex Male vs. female (ref.)	0.957 (0.539–1.698)	0.880	-	0.354
Recipient BMI (kg/m^2^)	1.018 (0.949–1.092)	0.619	-	0.715
Pre-existing recipient hypertension yes vs. no (ref.)	2.797 (0.386–20.272)	0.309	-	0.401
Pre-existing recipient diabetes yes vs. no (ref.)	2.044 (1.018–4.102)	0.044	-	0.827
Cause of ESRD	-	0.717	-	0.942
Time on dialysis (months)	0.999 (0.992–1.007)	0.833	-	0.376
Prior kidney transplantation ≥1 vs. 0 (ref.)	0.702 (0.278–1.772)	0.454	-	0.331
Donor type Postmortem vs. living donor (ref.)	3.121 (1.236–7.879)	0.016	-	0.774
Number HLA mismatch 4–6 vs. 0–3	1.814 (1.028–3.201)	0.040	-	0.504
Current PRA >20% vs. 0–20%	1.073 (0.148–7.780)	0.944	-	0.709
Cold ischemia time (hours)	1.060 (1.006–1.116)	0.028	-	0.427
Donor age (years)	1.052 (1.029–1.075)	<0.001	-	0.485
Donor sex Male vs. female (ref.)	0.567 (0.311–1.034)	0.064	-	0.140
NODAT yes vs. no (ref.)	3.163 (1.787–5.596)	<0.001	3.203 (1.451–7.072)	0.003
CMV DNAaemia yes vs. no (ref.)	1.331 (0.737–2.404)	0.344	-	0.443
Acute rejection within one year yes vs. no (ref.)	1.909 (1.024–3.558)	0.042	-	0.943
eGFR at month 3 (mL/min/1.73m^2^)	0.958 (0.941–0.976)	<0.001	-	0.851
eGFR at month 12 (mL/min/1.73m^2^)	0.941 (0.916–0.967)	<0.001	0.943 (0.915–0.971)	<0.001

Results are presented as hazard ratios (HR) with their 95% confidence interval (CI) and *p*-value of likelihood ratio test. For non-selected variables in multivariable analyses, *p*-value of score test is given. HR = hazard ratio; CI = confidence interval.

**Table 4:** Causes of death for slow and fast metabolizers.

	Slow Metabolizers (*n* = 12)	Fast Metabolizers (*n* = 15)
Cardiovascular	4 (33.3)	6 (40)
Infection	5 (41.7)	4 (26.7)
Tumor disease	2 (16.7)	-
Unknown	1 (8.3%)	5 (33.3)

**Table 5:** (a) Univariable Analysis: eGFR at month 12 and linear time-trends of eGFR (between months 12 and 60) by subgroup/marker. (b) Multivariable Analysis: eGFR at month 12 and linear time-trends of eGFR (between month 12 and 60) by subgroup/marker.

(**a**)				
	**Variable**	**B**	**95% CI**	***p***
Metabolizer type				
	Fast vs. slow (at month 12)	−3.54	−8.57 to 1.49	0.167
	Fast vs slow (time-trends)	−1.07	−2.10 to −0.05	0.040
R_Sex				
	Male vs. female (at month 12)	−16.21	−1.26 to −11.61	<0.001
	Male vs. female (time-trends)	0.49	−0.49 to 1.47	0.325
PreHypertension				
	No vs. yes (at month 12)	0.78	−9.63 to 11.19	0.883
	No vs. yes (time-trends)	1.26	−0.79 to 0.20	0.240
PreDiabetes				
	No vs. yes (at month 12)	4.52	−3.04 to 12.08	0.241
	No vs. yes (time-trends)	0.91	−0.68 to 2.51	0.262
Cause of ESRD				
	Cause of ESRD (at month 12)	-	-	0.010 *
	Diabetes vs. Hypertension (at month 12)	3.72	−15.38 to 22.82	0.703
	Polycystic kidney disease vs. Hypertension (at month 12)	8.30	−1.90 to 18.50	0.111
	Obstructive Nephropahty vs. Hypertension (at month 12)	16.20	4.91 to 27.48	0.005
	Glomerulonephritis vs. Hypertension (at month 12)	5.82	−3.12 to 14.76	0.202
	FSGS vs. Hypertension (at month 12)	7.14	−10.58 to 24.85	0.429
	Interstitial nephritis vs. Hypertension (at month 12)	11.40	−8.85 to 31.65	0.269
	Vasculitis vs. Hypertension (at month 12)	2.51	−16.28 to 21.32	0.792
	Other vs. Hypertension (at month 12)	16.81	7.14 to 26.48	0.001
	Cause of ESRD (time-trends)	-	-	0.998 *
PriorTx				
	No vs. yes (at month 12)	−8.67	−15.48 to −1.86	0.013
	No vs. yes (time-trends)	0.25	−1.13 to 1.63	0.719
DonorType				
	Postmortal vs. Living (at month 12)	−11.15	−16.40 to −5.90	<0.001
	Postmortal vs. Living ( time-trends)	0.47	−0.60 to 1.55	0.387
HLA Mismatch				
	0–3 vs. 4–6 (at month 12)	5.45	0.37 to 10.53	0.035
	0–3 vs. 4–6 (time-trends)	−0.21	−1.25 to 0.83	0.696
CurrentPRA				
	0–20 vs. >20 (at month 12)	−14.81	−32.19 to 2.57	0.095
	0–20 vs. >20 (time-trends)	−0.80	−4.15 to 2.54	0.638
D_Sex				
	Male vs. female (at month 12)	4.31	−0.47 to 9.09	0.077
	Male vs. female (time-trends)	−0.36	−1.32 to 0.62	0.470
NODAT				
	No vs. yes (at month 12)	6.50	1.31 to 11.69	0.014
	No vs. yes (time-trends)	0.52	−0.56 to 1.60	0.342
CMV DNAaemia				
	No vs. yes (at month 12)	4.46	−0.74 to 9.66	0.093
	No vs. yes (time-trends)	−0.26	−1.32 to 0.80	0.629
Acute rejection 1 year post RTx				
	No vs. yes (at month 12)	16.23	10.34 to 22.13	<0.001
	No vs. yes (time-trends)	0.09	−1.18 to 1.35	0.893
R-Age (years)				
	R-Age (at month 12)	−0.47	−0.63 to −0.32	<0.001
	R-Age (time-trends)	−0.004	−0.013 to 0.005	0.415
R-BMI				
	R-BMI (at month 12)	−1.12	−1.67 to −0.57	<0.001
	R-BMI (time-trends)	−0.008	−0.027 to 0.011	0.405
Time on Dialysis (month)				
	Time on Dialysis (at month 12)	−0.05	−0.11 to 0.01	0.112
	Time on Dialysis (time-trends)	−0.012	−0.008 to 0.006	0.743
CIT (hours)				
	CIT (at month 12)	−0.43	−0.86 to 0.004	0.052
	CIT (time-trends)	−0.014	−0.064 to 0.034	0.565
D_Age (years)				
	D-Age (at month 12)	−0.65	−0.78 to −0.52	<0.001
	D-Age (time-trends)	−0.006	−0.015 to 0.002	0.152
(**b**)				
	**Variable**	**Estimate**	**95% CI**	***p***
At month 12				
	Metabolizer type: fast vs. slow	−2.48	−6.47 to 1.51	0.222
	D_Age (years)	−0.60	−0.71 to −0.48	<0.001
	R_Sex: male vs. female	−12.27	−15.75 to −8.79	<0.001
	Donor type: postmortem vs. living	−10.03	−13.94 to −6.12	<0.001
	R_BMI (kg/m^2^)	−0.58	−1.03 to −0.14	0.010
	PreHypertension: no vs. yes			N/S: 0.051
	PreDiabetes: no vs. yes			N/S: 0.914
	Cause of ESRD Diabetes vs. Hypertension Polycystic kidney disease vs. Hypertension Obstructive Nephropathy vs. Hypertension Glomerulonephritis vs. Hypertension FSGS vs. Hypertension Interstitial nephritis vs. Hypertension Vasculitis vs. Hypertension Other vs. Hypertension	10.79 4.72 11.34 2.83 5.16 2.23 −0.31 11.26	−2.08 to 23.65 −2.57 to 12.02 3.11 to 19.56 −3.53 to 9.19 −6.98 to 17.31 −14.60 to 19.06 −13.54 to 12.93 4.30 to 18.22	0.010 * 0.100 0.204 0.007 0.382 0.404 0.794 0.964 0.002
	PriorTx: no vs yes			N/S: 0.225
	HLAMismatch: 0–3 vs. 4–6			N/S: 0.713
	CurrentPRA: 0–20 vs. >20			N/S: 0.272
	D_Sex: male vs. female			N/S: 0.107
	NODAT: no vs. yes			N/S: 0.995
	CMV DNAaemia: no vs. yes			N/S: 0.417
	Acute rejection 1 year post RTx: no vs. yes	14.00	9.64 to 18.36	<0.001
	R_Age (years)			N/S: 0.495
	Time Dialysis (months)			N/S: 0.112
	CIT (hours)			N/S: 0.771
Time trends				
	Metabolizer type: fast vs. slow	−1.07	−2.05 to −0.09	0.032
	D_Age (years)			N/S: 0.121
	R_Sex: male vs. female			N/S: 0.240
	Donor Type: postmortem vs. living			N/S: 0.666
	R_BMI (kg/m^2^)			N/S: 0.810
	PreHypertension: no vs. yes			N/S: 0.366
	PreDiabetes: no vs. yes			N/S: 0.354
	Cause of ESRD			N/S: 0.997 *
	PriorTx: no vs. yes			N/S: 0.635
	HLAMismatch: 0–3 vs. 4–6			N/S: 0.299
	CurrentPRA: 0–20 vs. >20			N/S: 0.708
	D_Sex: male vs. female			N/S: 0.293
	NODAT: no vs. yes			N/S: 0.368
	CMV DNAaemia: no vs. yes			N/S: 0.519
	Acute rejection1 year post RTx: no vs. yes			N/S: 0.913
	R_Age (years)			N/S: 0.332
	Time Dialysis (months)			N/S: 0.840
	CIT (hours)			N/S: 0.400

* *p*-value of F-test (global test).

**Table 6:** Cox regression model for rejection-free survival. Univariable and multivariable analyses of rejection-free survival using Cox regression. Results are presented as hazard ratios (HR) with their 95% confidence interval (CI) and *p*-value of likelihood ratio test. For non-selected variables in multivariable analyses, *p*-value of score test is given.

Parameters	Univariable		Multivariable	
	HR (95% CI)	*p*-Value	HR (95% CI)	*p*-Value
Fast metabolizers vs. slow metabolizers (ref.)	1.536 (1.034–2.282)	0.035	1.622 (1.085–2.424)	0.020
Age (years)	0.996 (0.981–1.010)	0.547	-	0.615
Recipient sex Male vs. female (ref.)	1.432 (0.943–2.176)	0.092	-	0.122
Recipient BMI (kg/m^2^)	1.057 (1.007–1.110)	0.026	1.073 (1.021–1.128)	0.006
Pre-existing recipient hypertension yes vs. no (ref.)	1.379 (0.507–3.751)	0.529	-	0.695
Pre-existing recipient diabetes yes vs. no (ref.)	1.032 (0.564–1.887)	0.919	-	0.716
Cause of ESRD	-	0.999	-	0.998
Time on dialysis (months)	1.000 (0.996–1.005)	0.862	-	0.746
Prior kidney transplantation ≥1 vs. 0 (ref.)	1.632 (0.999–2.665)	0.051	1.850 (1.109–3.087)	0.027
Donor type Postmortem vs. living donor (ref.)	0.765 (0.498–1.174)	0.220	-	0.249
Number HLA mismatch 4–6 vs. 0–3	1.043 (0.683–1.593)	0.845	-	0.905
Current PRA >20% vs. 0–20%	1.033 (0.255–4.189)	0.964	-	0.830
Cold ischaemia time (hours)	0.986 (0.948–1.026)	0.489	-	0.620
Donor age (years)	1.002 (0.989–1.014)	0.788	-	0.846
Donor sex Male vs. female (ref.)	0.936 (0.629–1.391)	0.742	-	0.632

HR = hazard ratio; CI = confidence interval.

**Table 7:** Frequencies of acute rejections and subtype analysis of the first rejection after RTx within five years after transplantation. Fast metabolizers showed increased frequencies of acute biopsy-proven rejections as compared to slow metabolizers. The *p*-value from Fisher’s exact test is given.

	Slow Metabolizers (*n* = 253)	Fast Metabolizers (*n* = 148)	*p*-Value
Type of acute rejection			0.084
No rejection	199 (78.7)	103 (69.6)	
Humoral	9 (3.6)	10 (6.8)	
Mixed	6 (2.4)	10 (6.8)	
Cellular	15 (5.9)	12 (8.1)	
Borderline	24 (9.5)	13 (8.8)	

## Supplementary Materials

The following are available online at https://www.mdpi.com/2077-0383/8/5/587/s1, Table S1: The average C/D ratio of month one and six for 50 randomly selected patients did not differ from the 3-month C/D ratio, suggesting that 3-month C/D ratio strongly correlated with the average C/D ratio during month one and six. P-value of Mann-Whitney U test is given, Table S2: Categorization of slow and fast Tac metabolizers was similar when applying the 3-month C/D ratio or the average C/D ratio of month one and six (*p* = 1.000, Fisher’s exact test).
