# Correction: Fast Tac Metabolizers at Risk—It is Time for a C/D Ratio Calculation. *J. Clin. Med.* 2019, *8*, 587

**DOI:** 10.3390/jcm8111870

**Published:** 2019-11-04

**Authors:** Katharina Schütte-Nütgen, Gerold Thölking, Julia Steinke, Hermann Pavenstädt, René Schmidt, Barbara Suwelack, Stefan Reuter

**Affiliations:** 1 Department of Medicine D, Division of General Internal Medicine, Nephrology and Rheumatology, University Hospital of Münster, 48149 Münster, Germany; katharina.schuette-nuetgen@gmx.de (K.S.-N.); gerold.thoelking@ukmuenster.de (G.T.); j_steinke@ymail.com (J.S.); hermann.pavenstaedt@ukmuenster.de (H.P.); barbara.suwelack@ukmuenster.de (B.S.); 2 Institute of Biostatistics and Clinical Research, University Hospital of Münster, 48149 Münster, Germany; rene.schmidt@ukmuenster.de

The authors wish to make the following corrections to this paper [1].

The authors made an error regarding the rejection-free survival curve in Figure 4A. Figure 4 needs to be corrected.


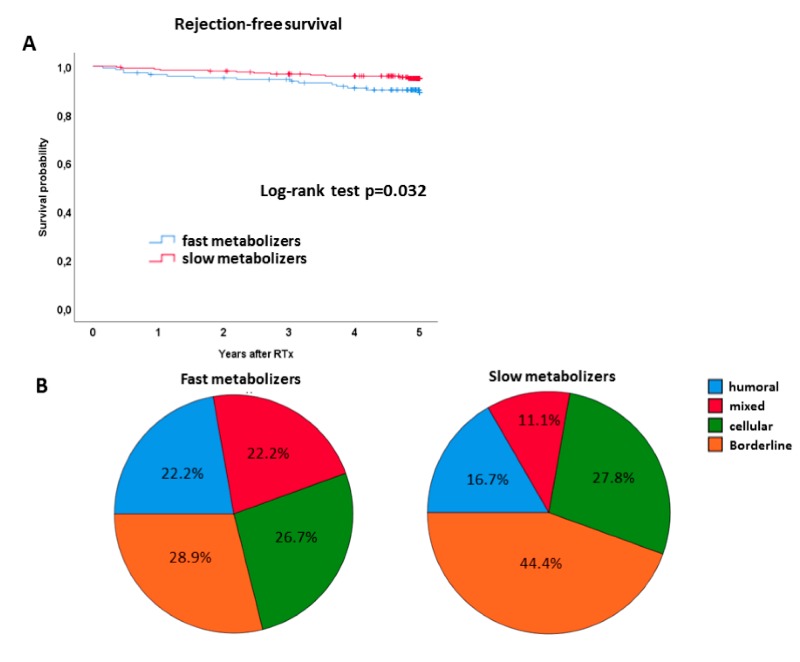


should be replaced with


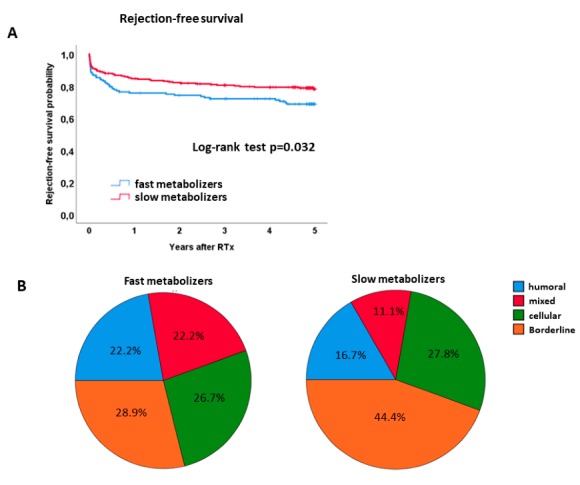


The authors apologize to the readers for any inconvenience caused by these changes. It is important to state that this correction do not affect our study’s results and involve no changes or modifications in the original data supporting our results. The original manuscript will remain online on the article webpage, with reference to this Correction.
