# Supplementary material for: Fast Tac Metabolizers at Risk—It is Time for a C/D Ratio Calculation
Source: J Clin Med. 2019 Apr 28;8(5):587. doi: 10.3390/jcm8050587 (PMC6572069; doi:10.3390/jcm8050587)
Supplement: Supplementary file 1 [file jcm-08-00587-s001.pdf]

Journal of
Clinical Medicine

Supplementary Material

	3-month C/D ratio	Average C/D ratio (month 1 and 6)	p-value
**Median**	1,29	1,19	0.765
**25% percentile**	0,87	0,82	
**75% percentile**	2,00	1,96	

Supplemental Table S1. The average C/D ratio of month one and six for 50 randomly selected patients did not differ from the 3-month C/D ratio, suggesting that 3-month C/D ratio strongly correlated with the average C/D ratio during month one and six. P-value of Mann-Whitney U test is given.

	**Average C/D ratio (month 1 and 6)**	
**3-month C/D ratio**	Slow metabolizer	Fast metabolizer
Slow metabolizer	24	1
Fast metabolizer	1	24

Supplemental Table S2. Categorization of slow and fast Tac metabolizers was similar when applying the 3-month C/D ratio or the average C/D ratio of month one and six (p=1.000, Fisher's exact test).
